# Supplementary material for: Gene therapy via canalostomy approach preserves auditory and vestibular functions in a mouse model of Jervell and Lange-Nielsen syndrome type 2
Source: Nat Commun. 2021 Jan 29;12:697. doi: 10.1038/s41467-020-20808-7 (PMC7846845; doi:10.1038/s41467-020-20808-7)
Supplement: Supplementary file 1 — Supplementary Information [file 41467_2020_20808_MOESM1_ESM.pdf]

Gene therapy via canalostomy approach preserves auditory and vestibular functions in a mouse model of Jervell and Lange-Nielsen syndrome type 2

Xuewen Wu[1,2], Li Zhang[2, 3], Yihui Li[4], Wenjuan Zhang[3], Jianjun Wang[2], Cuiyun Cai[1,2], Xi Lin[2*]

***Correspondence author:** Xi Lin, PhD (xlin2@emory.edu).

Supplementary Information

(Contains Supplementary Figures 1-9 with Legends)

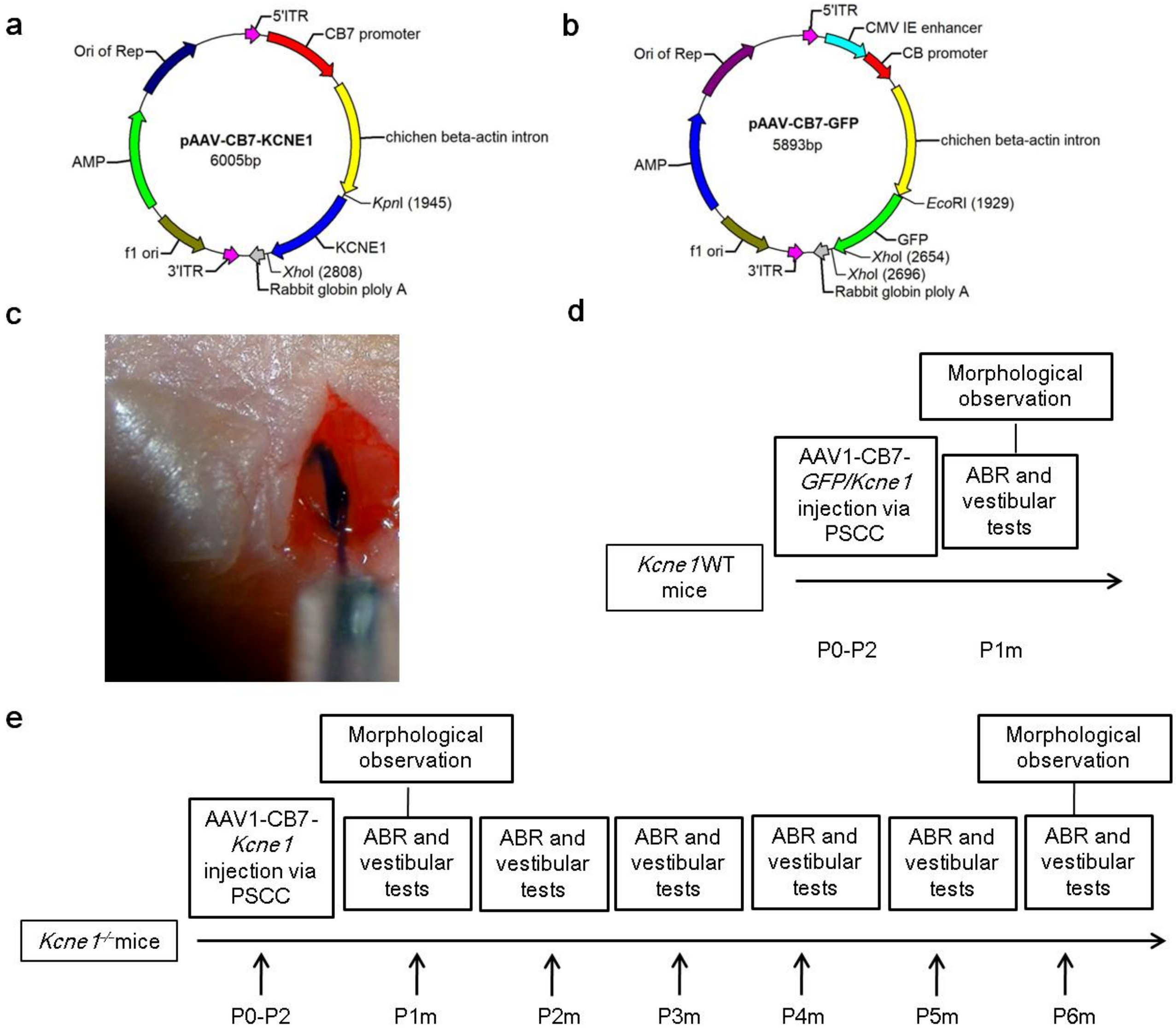

Supplementary Figure 1. Basic information of viral plasmids and design of experimental protocols. **a** Schematic map of plasmid *Kcne1*. **b** Schematic map of plasmid *GFP*. **c** A picture showing the location of posterior semicircular canal (PSCC) injection in neonatal mice. **d, e** Diagram showing the protocol for gene delivery and functional evaluations in *Kcne1*$^{-/-}$ and WT mice.

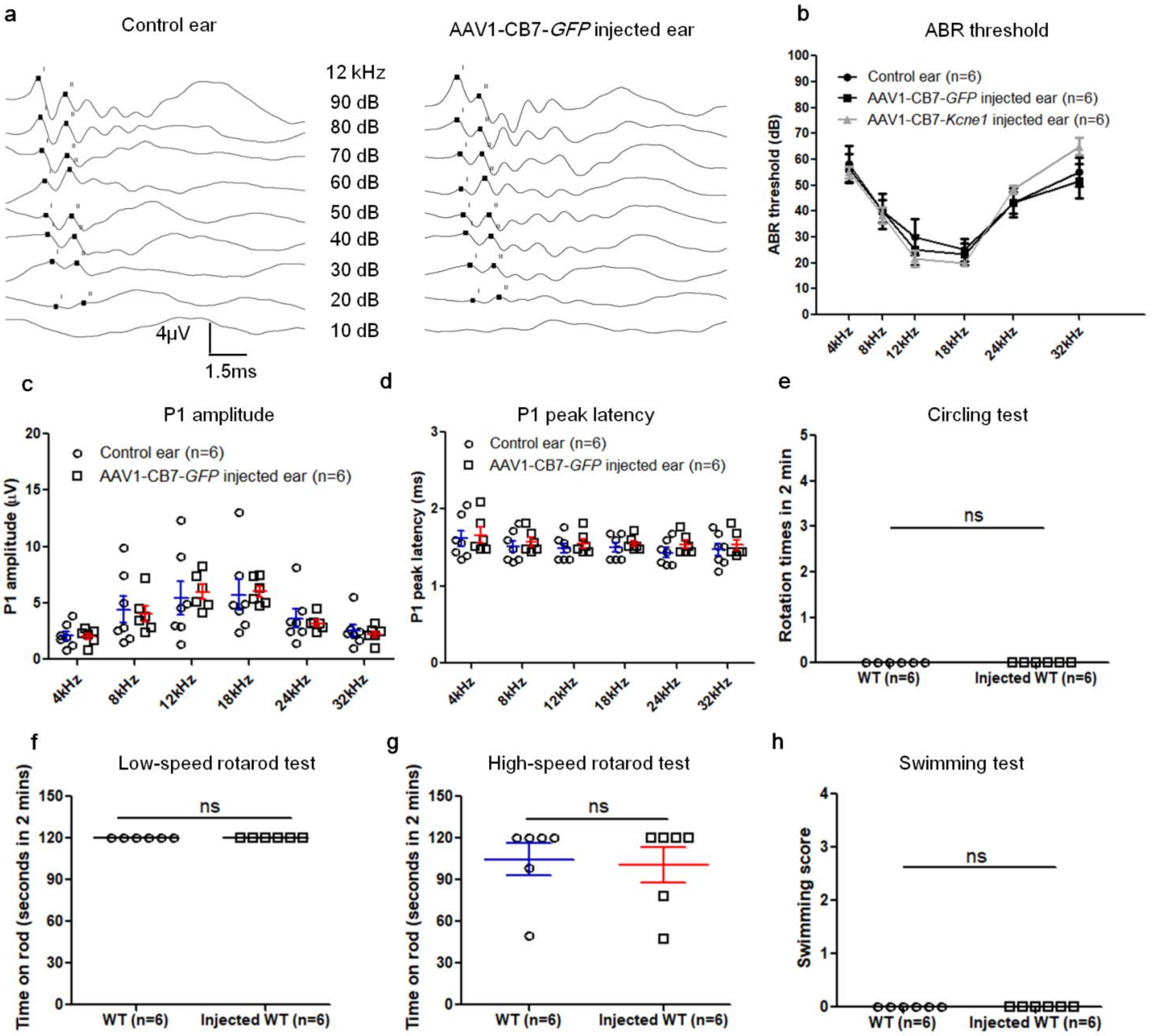

Supplementary Figure 2. Auditory brainstem response (ABR) and vestibular functions examined at P30 after injection of AAV1-CB7-*GFP* or AAV1-CB7-*Kcne1* via posterior semicircular canal (PSCC) approach at P0–P2 in the WT mice. a Representative series of ABR waveforms evoked by different intensities of tone burst at 12 kHz from control and injected ears. Sound pressure level is shown in dB SPL and indicated by the right of data traces. **b** ABR thresholds (mean ± SEM). Animal groups received either AAV1-CB7-*GFP* or AAV1-CB7-*Kcne1* injections are indicated by figure legends (P>0.05 at all frequencies, two-sided student's *t* tests). Source data are provided as a Source Data file. **c** Peak amplitude of the ABR wave 1 (P1) measured from tone-burst responses at 4–32 kHz. Data are shown as mean ± SEM (P>0.05 at all frequencies, two-sided student's *t* tests). Source data are provided as a Source Data file. **d** P1 latencies of ABR waveforms measured at responses evoked at 4–32 kHz. Data are shown as mean ± SEM (P>0.05 at all frequencies, two-sided student's *t* tests). Source data are provided as a Source Data file. **e–h** Comparisons of the vestibular functional test results in injected and un-injected WT mice. Different groups of animals are indicated by figure legends. Data are shown as mean ± SEM (P>0.05 in all comparisons, two-sided student's *t* tests). Source data are provided as a Source Data file.

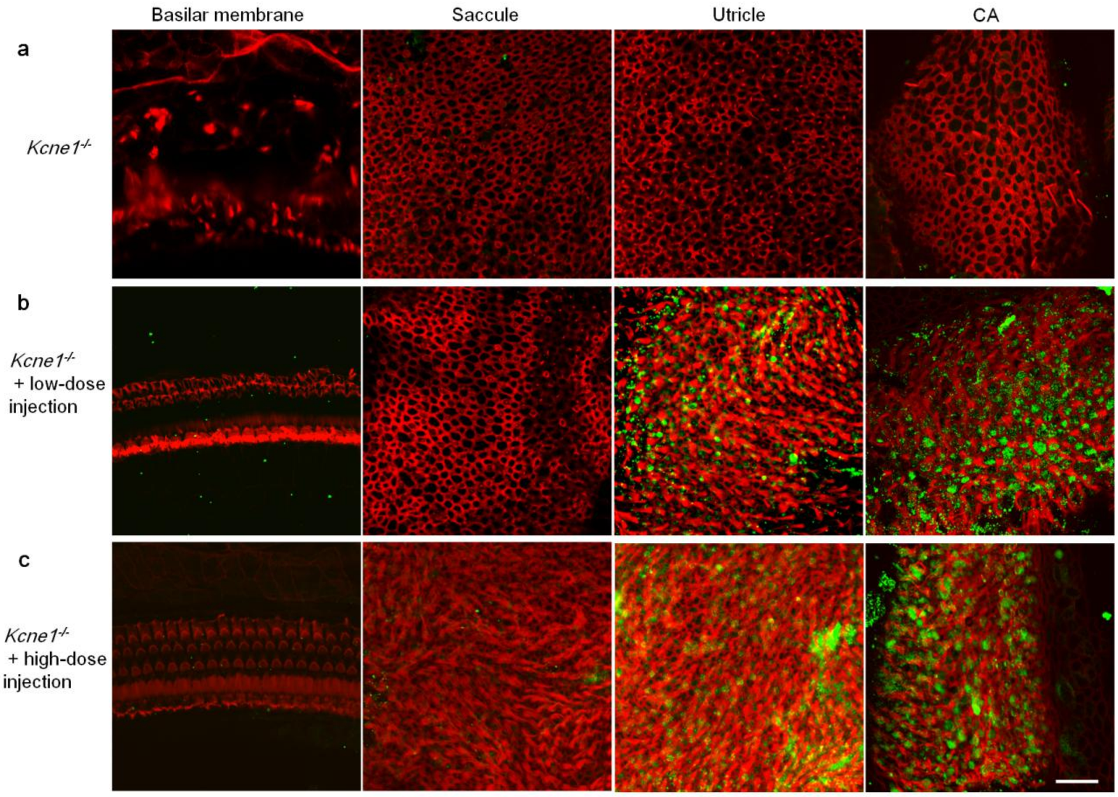

Supplementary Figure 3. *Kcne1* expression in cochlear hair cells (HCs) and vestibular end-organs in untreated, low- and high-dose treated *Kcne1*$^{-/-}$ mice examined at P30 (n=4 in each group). a *Kcne1* expressions in the basilar membrane, saccule, utricle, or crista ampullaris (CA) of in untreated *Kcne1*$^{-/-}$ mice. **b** Expressions of *Kcne1* was detected in the CA and utricle in the low-dose treated ears of *Kcne1*$^{-/-}$ mice. No KCNE1 signal was detected in saccule and cochlear HCs. **c** Expressions of *Kcne1* was found in the CA and utricle in the high-dose treated ears of the *Kcne1*$^{-/-}$ mice. No virally mediated *Kcne1* expression was detected in the cochlear HCs. Red: phalloidin; Green: KCNE1. Scale bar is 100 μm and applies to all panels.

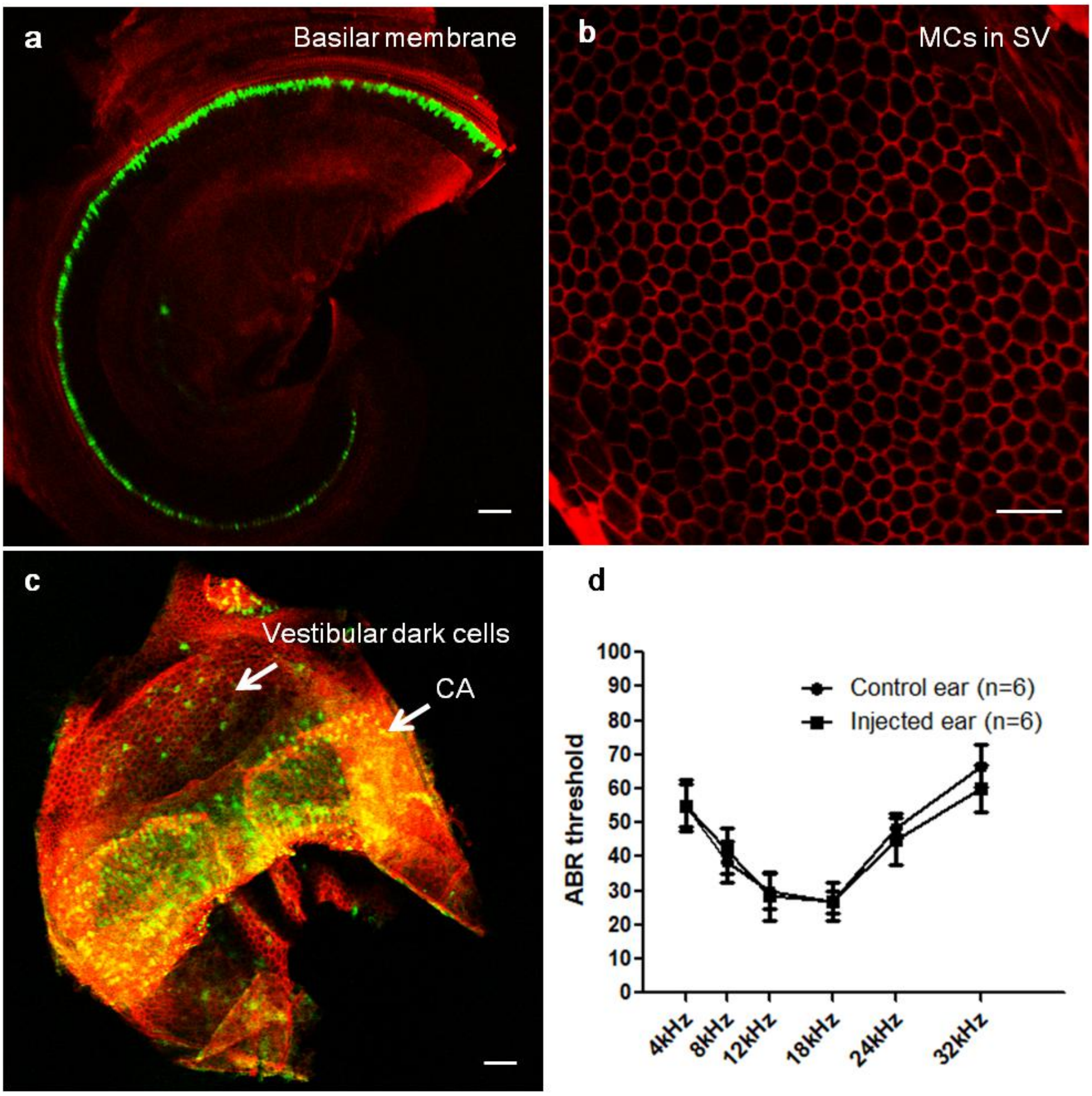

Supplementary Figure 4. Confocal immunofluorescent images and auditory brainstem response (ABR) tests in adult WT mice seven days after AAV1-CB7-*GFP* delivery via posterior semicircular canal (PSCC) injections at P30 (n=6 in each group). a A representative confocal image of the region of the middle turn basilar membrane. Extensive *GFP* expression was found in the IHCs. **b** Confocal images of the SV showing no *GFP* expression in the stria vascularis (SV). **c** Confocal images of the crista ampullaris (CA) and vestibular dark cells. *GFP* expression was found in various types of vestibular cells in the CA and in the vestibular dark cells. **d** ABR thresholds (mean ± SEM) measured from the control and injected adult WT mice (indicated by figure legends) 7 days after AAV1-CB7-*GFP* delivery via PSCC injections at P30 ($P>0.05$ at all frequencies, two-sided student's *t* tests). Data are shown as mean ± SEM. Source data are provided as a Source Data file. Red: phalloidin; Green: GFP. Scale bar is 100 µm and applies to all panels.

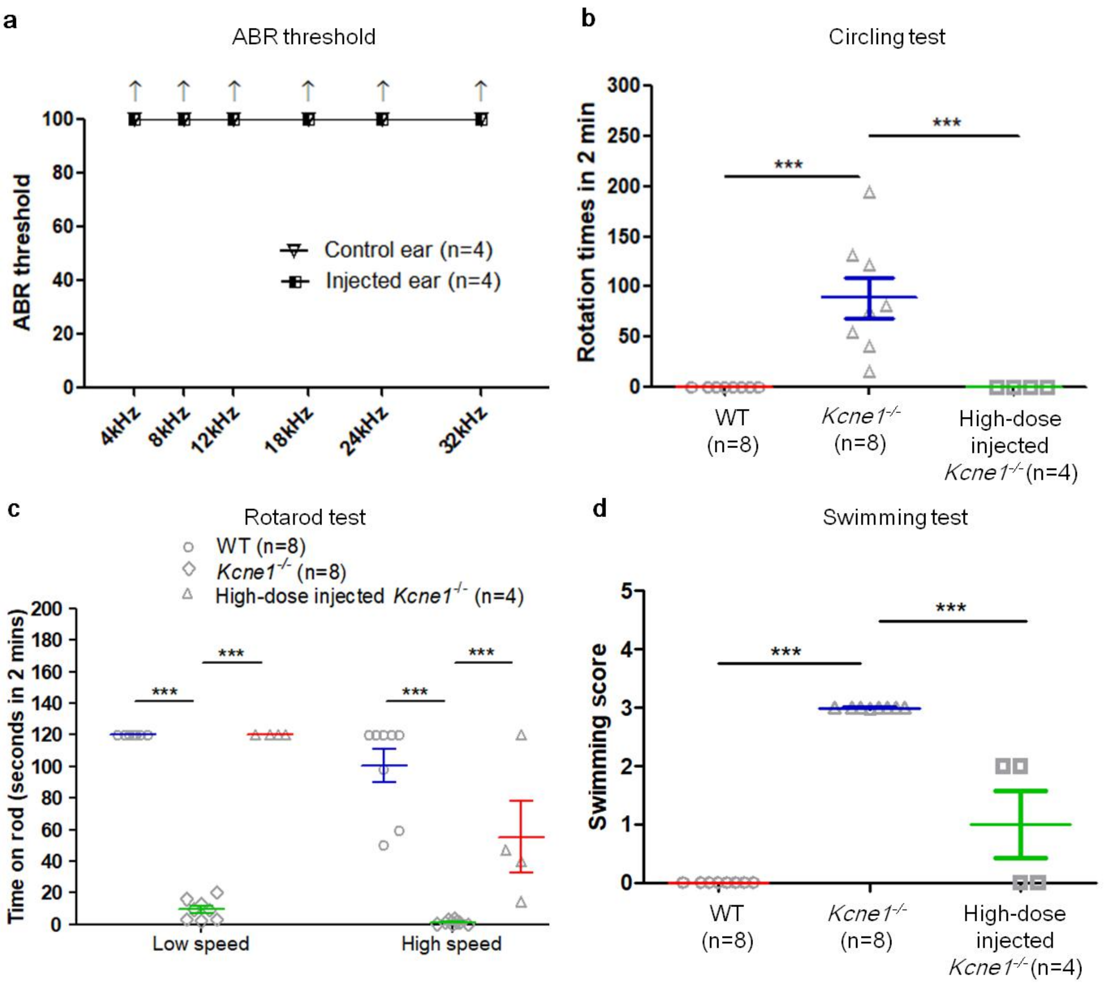

Supplementary Figure 5. Auditory brainstem responses (ABR) tests and vestibular functional assessments at P30 after a high dosage of AAV1-CB7-*Kcne1* injection through the posterior semicircular canal (PSCC) route at P3 in the *Kcne1*$^{-/-}$ mice. a Comparison of the ABR thresholds from the control and injected mice (P>0.05 at all frequencies, two-sided student's *t* tests). Data are shown as mean ± SEM. Source data are provided as a Source Data file. **b** Results of circling tests from WT, untreated, and high-dose-treated *Kcne1*$^{-/-}$ mice (p<0.0001 in all comparisons, two-sided student's *t* tests). Data are shown as mean ± SEM. Source data are provided as a Source Data file. **c** Results of low-speed and high-speed rotarod tests from WT, untreated, and high-dose-treated *Kcne1*$^{-/-}$ mice (p<0.0001 in all comparisons, two-sided student's *t* tests). Data are shown as mean ± SEM. Source data are provided as a Source Data file. **d** Results of swimming tests from WT, untreated, and high-dose-treated *Kcne1*$^{-/-}$ mice (p<0.0001 in all comparisons, two-sided student's *t* tests). Data are shown as mean ± SEM. Source data are provided as a Source Data file. Data obtained from different animal groups are indicated by figure legends.

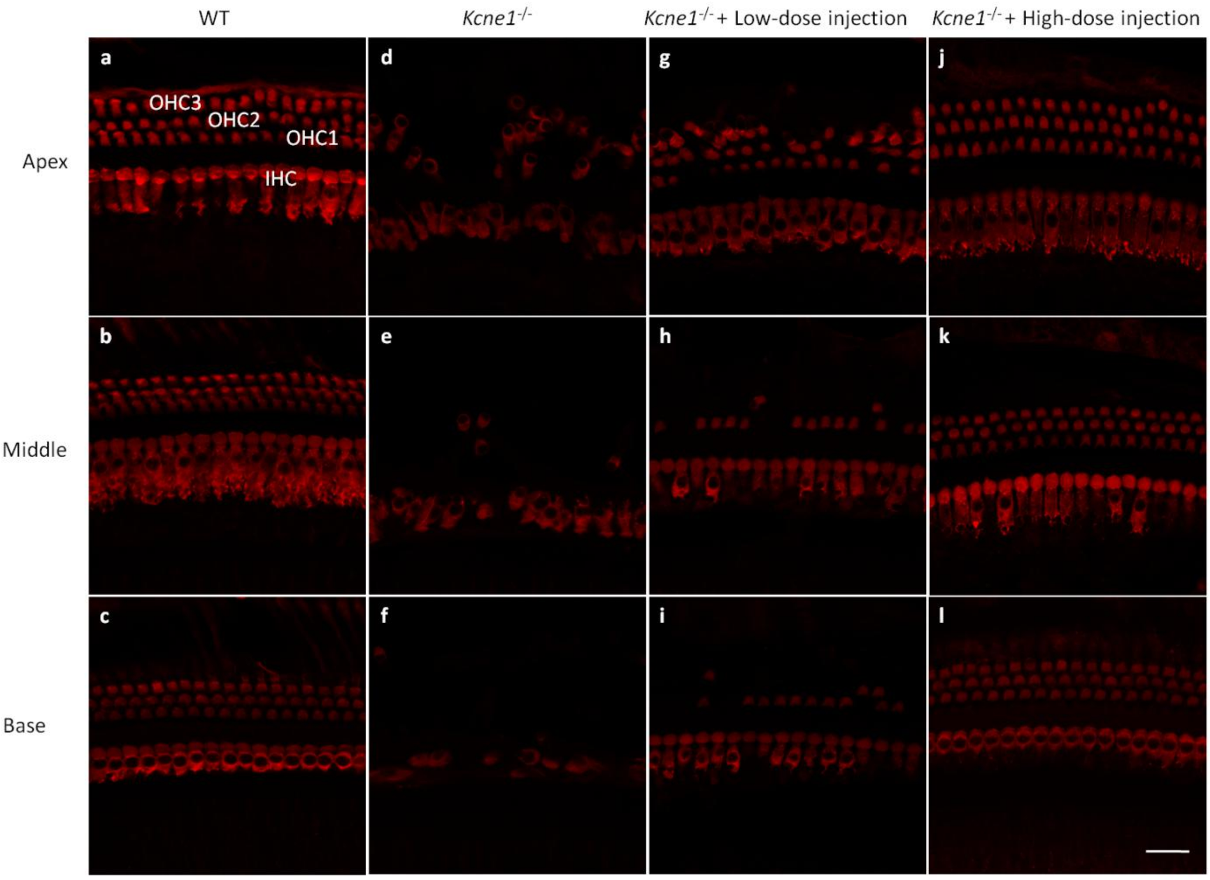

Supplementary Figure 6. Confocal immunofluorescent images of cochlear hair cells (HCs) bodies in WT ears, untreated, low- and high-dose treated ears of *Kcne1*$^{-/-}$ mice at P30 (n=4 in each group). a-c In WT cochleae, the shape and arrangement of inner hair cells (IHCs) and outer hair cells (OHCs) bodies displayed their normal patterns in apex, middle and base turns. **d-f** both types of cochlear HCs were severely degenerated in all turns in untreated ears of *Kcne1*$^{-/-}$ mice. **g-i** In low-dose-treated ears of *Kcne1*$^{-/-}$ mice, the number and shape of IHCs were apparently normal in all turns. However, most OHCs appeared to be degenerated in the middle and basal turns. **j-l** After injections with the high-dosage of AAV1-CB7-*Kcne1*, all IHCs and OHCs appeared to be normal in all turns. Red: Myo7a. Scale bar is 50 µm and applies to all panels.

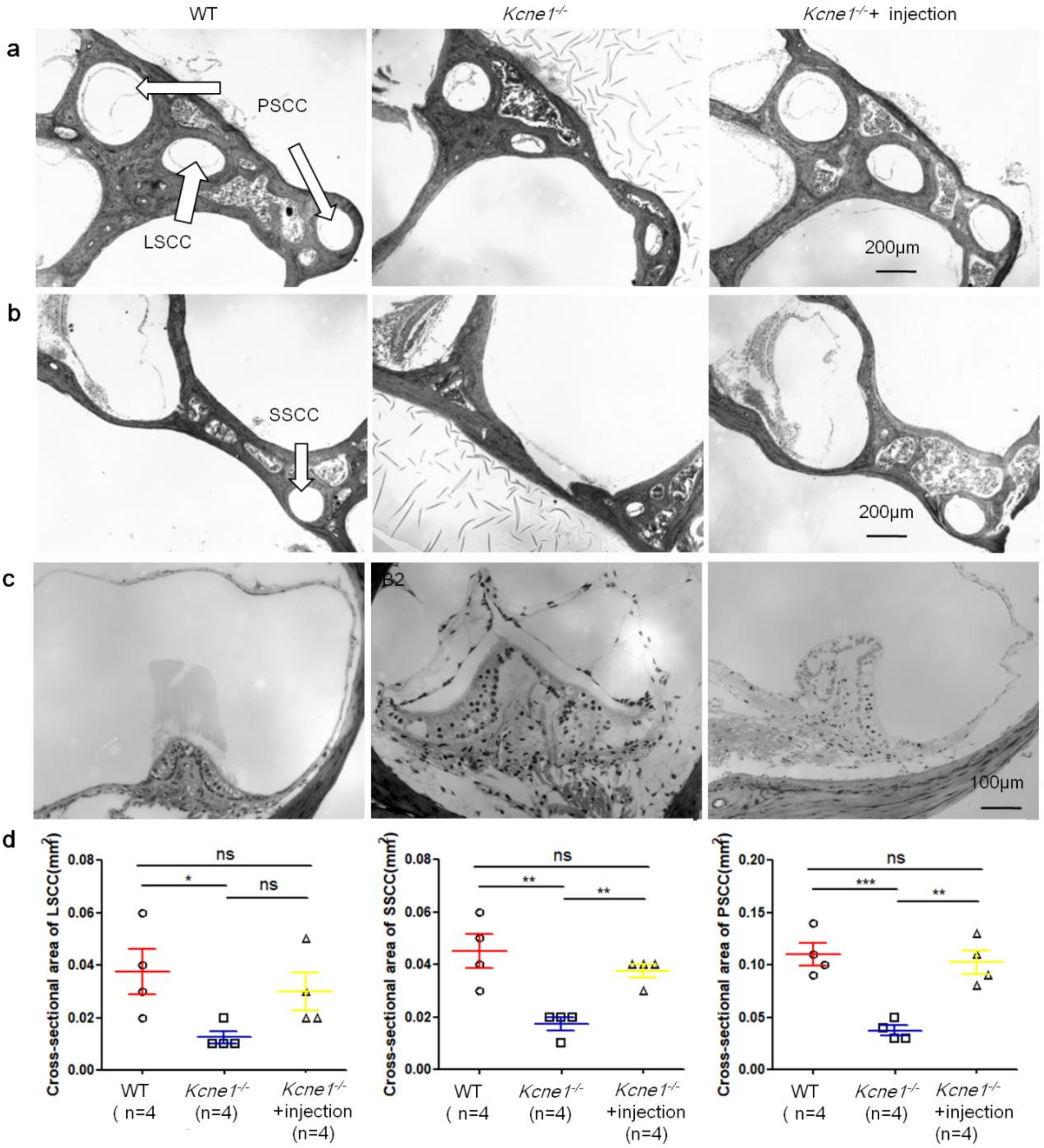

Supplementary Figure 7. Cross-sections of the semicircular canals and the crista ampullaris (CA) in WT, untreated, and high-dose-treated *Kcne1$^{-/-}$* ears obtained at P30 (n=4 in each group). a Cross section of the lateral semicircular canal (LSCC) and posterior semicircular canal (PSCC) in different groups as indicated by figure legends. **b** Cross-section of the superior semicircular canal (SSCC) as indicated by figure legends. **c** Sections of the CA and vestibular wall in different groups. Untreated *Kcne1$^{-/-}$* ears exhibited a collapsed vestibular wall surrounding the crista (middle panel). The vestibular wall returned to its normal position in treated ears of *Kcne1$^{-/-}$* mice (right panel). **d** Quantitative analysis of the cross-sectional areas of LSCC, PSCC and SSCC in WT, untreated, and high-dose-treated *Kcne1$^{-/-}$* ears (n=4 in each group; p=0.03 for LSCC, p=0.007 for SSCC, and p=0.0009 for PSCC in comparisons between WT ears and untreated *Kcne1$^{-/-}$* ears; p=0.058 for LSCC, p=0.001 for SSCC, and p=0.002 for PSCC in comparisons between high-dose-treated *Kcne1$^{-/-}$* ears and untreated *Kcne1$^{-/-}$* ears; two-sided student's *t* tests). Data are shown as mean ± SEM. Source data are provided as a Source Data file.

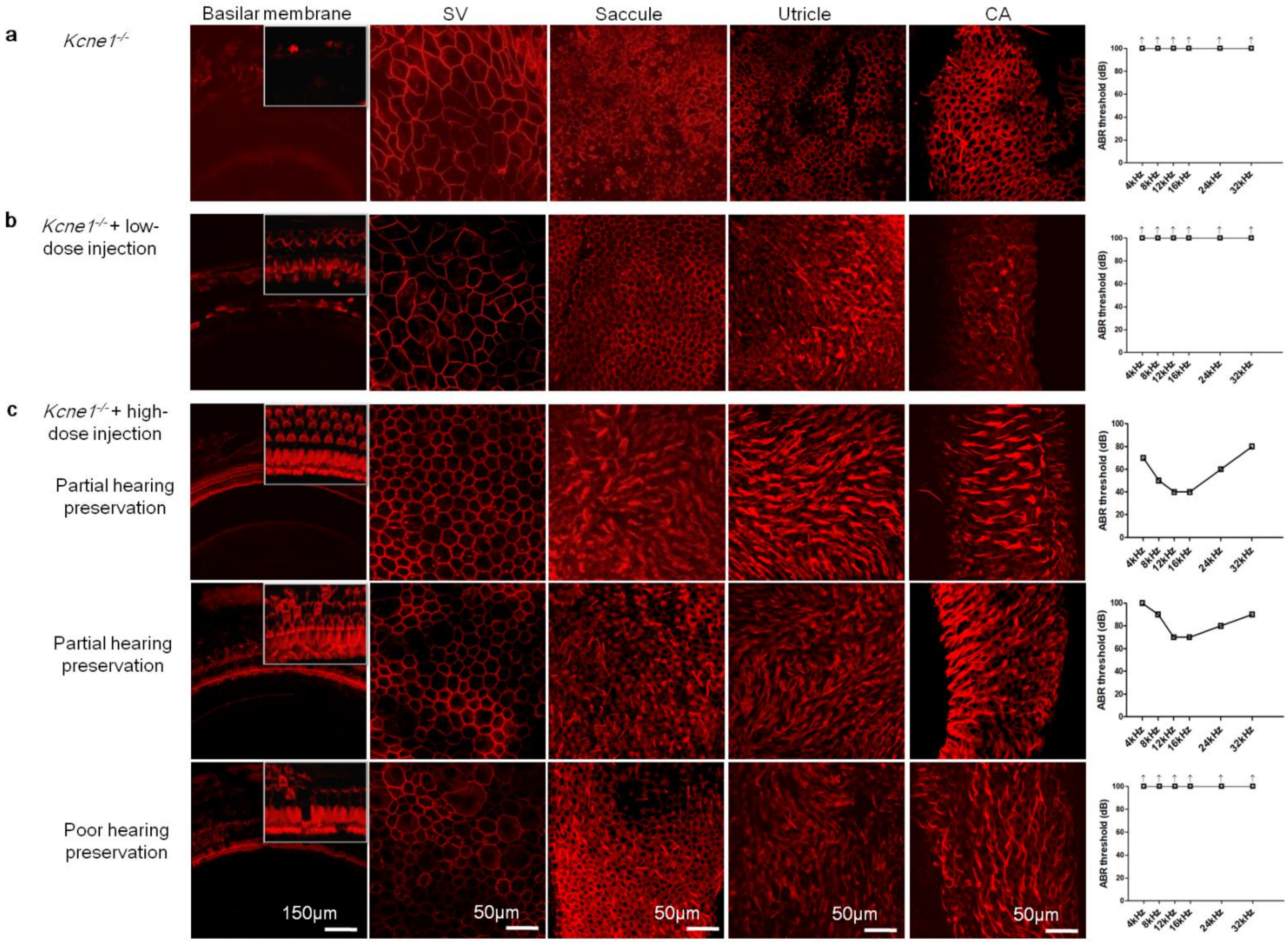

Supplementary Figure 8. Comparison of morphologies of the membranous labyrinths (from whole-mount preparations) and representative tone burst evoked auditory brainstem response (ABR) thresholds from untreated, low- and high-dose treated *Kcne1*$^{-/-}$ mice at P6m (n=6 in each group). **a** All of the ciliary bundles of cochlear and vestibular hair cells (HCs) were missing, and the morphology of marginal cells (MCs) was also abnormal in untreated *Kcne1*$^{-/-}$ mice with undetectable ABRs at all frequencies. **b** Only a few ciliary bundles of vestibular HCs in the utricle and crista ampullaris (CA) were normal, and cellular degeneration was observed in all cochlear HCs, MCs, and vestibular HCs in the saccule in the low-dosage group with undetectable ABRs at all frequencies. **c** In high-dosage group, the morphologies of whole-mount inner ear tissues obtained from two *Kcne1*$^{-/-}$ mice with partial hearing preservation were apparently normal at P6m (top row), though different degrees of cellular degeneration appeared in cochlear HCs, MCs and vestibular HCs in mice with partial hearing preservation (middle row) and with poor hearing preservation (bottom row). Red: phalloidin.

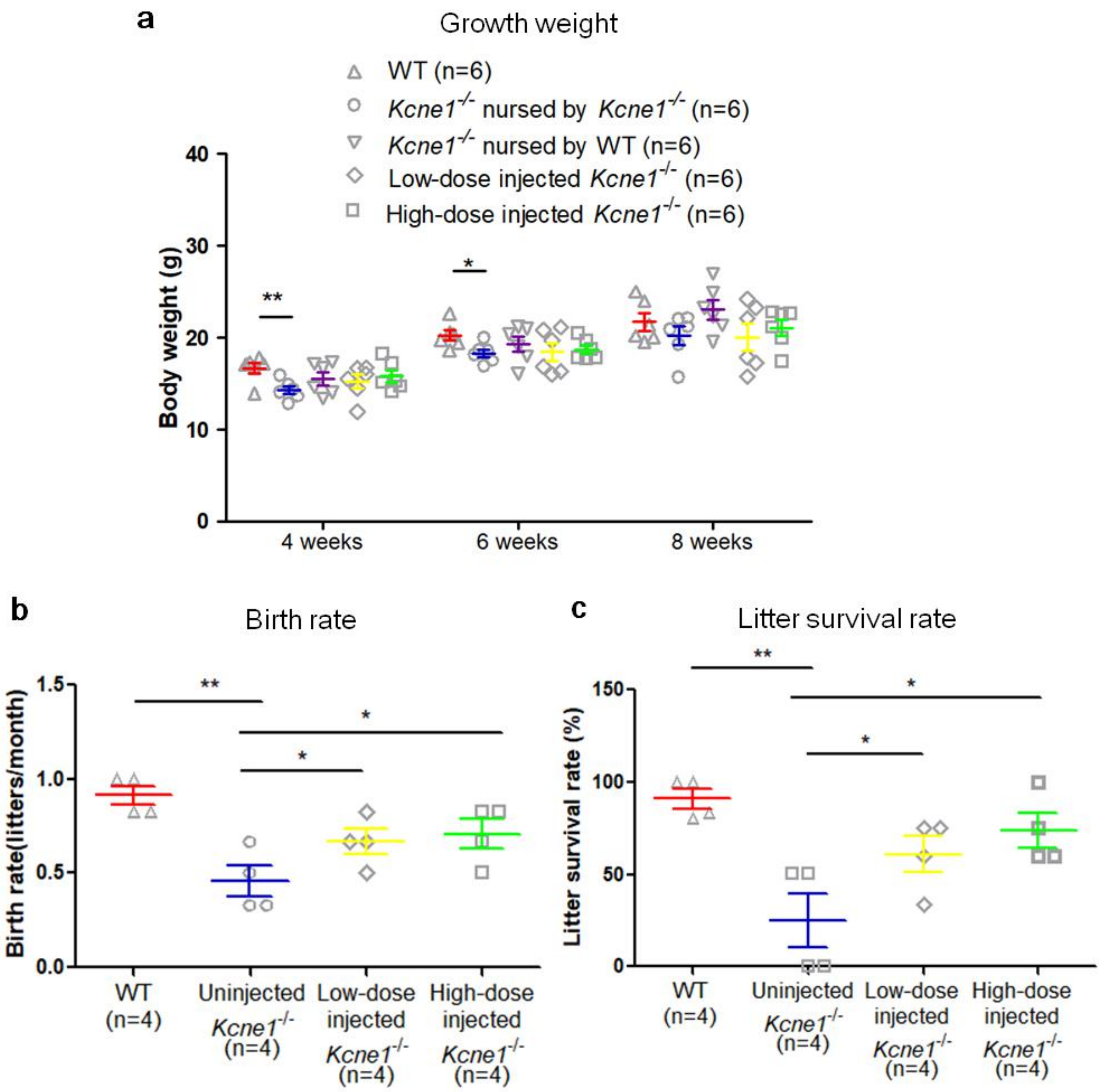

Supplementary Figure 9. Assessments of secondary outcomes after AAV1-CB7-*Kcne1* injection at P0–P2 in the *Kcne1*$^{-/-}$ mice. a Weight growth of mice at four, six, and eight weeks of age among WT mice, untreated *Kcne1*$^{-/-}$ mice nursed by WT or *Kcne1*$^{-/-}$ mice, low- and high-dose treated *Kcne1*$^{-/-}$ mice who are nursed by WT mothers (p=0.007 at 4 weeks, p=0.02 at 6 weeks, comparison between WT mice and untreated *Kcne1*$^{-/-}$ mice nursed by *Kcne1*$^{-/-}$ mice; p>0.05 in other comparisons; two-sided student's *t* tests). Data are shown as mean ± SEM. Source data are provided as a Source Data file. **b** Birth rates for the offspring among WT, untreated, low- and high-dose treated *Kcne1*$^{-/-}$ mice (p=0.003 in WT group, p=0.035 in high-dosage group and P=0.047 in low-dosage group, comparing to the untreated group, two-sided student's *t* tests). Data are shown as mean ± SEM. Source data are provided as a Source Data file. **c** Offspring survival rates among WT, untreated, low- and high-dose treated *Kcne1*$^{-/-}$ mice (p=0.005 in WT group, p=0.043 in low-dosage group and p=0.017 in high-dosage group, compared to untreated group, two-sided student's *t* tests). Data are shown as mean ± SEM. Source data are provided as a Source Data file.
