# Supplementary material for: Gene therapy via canalostomy approach preserves auditory and vestibular functions in a mouse model of Jervell and Lange-Nielsen syndrome type 2
Source: Nat Commun. 2021 Jan 29;12:697. doi: 10.1038/s41467-020-20808-7 (PMC7846845; doi:10.1038/s41467-020-20808-7)
Supplement: Supplementary file 9 — Reporting Summary [file 41467_2020_20808_MOESM9_ESM.pdf]

nature research

Corresponding author(s): xi lin

Last updated by author(s): Nov 8, 2020

Reporting Summary

Nature Research wishes to improve the reproducibility of the work that we publish. This form provides structure for consistency and transparency in reporting. For further information on Nature Research policies, see Authors & Referees and the Editorial Policy Checklist .

Statistics

For all statistical analyses, confirm that the following items are present in the figure legend, table legend, main text, or Methods section.

n/a	Confirmed	
☐	☒	The exact sample size (*n*) for each experimental group/condition, given as a discrete number and unit of measurement
☐	☒	A statement on whether measurements were taken from distinct samples or whether the same sample was measured repeatedly
☐	☒	The statistical test(s) used AND whether they are one- or two-sided<br>*Only common tests should be described solely by name; describe more complex techniques in the Methods section.*
☐	☒	A description of all covariates tested
☐	☒	A description of any assumptions or corrections, such as tests of normality and adjustment for multiple comparisons
☐	☒	A full description of the statistical parameters including central tendency (e.g. means) or other basic estimates (e.g. regression coefficient) AND variation (e.g. standard deviation) or associated estimates of uncertainty (e.g. confidence intervals)
☐	☒	For null hypothesis testing, the test statistic (e.g. *F*, *t*, *r*) with confidence intervals, effect sizes, degrees of freedom and *P* value noted<br>*Give P values as exact values whenever suitable.*
☐	☒	For Bayesian analysis, information on the choice of priors and Markov chain Monte Carlo settings
☐	☒	For hierarchical and complex designs, identification of the appropriate level for tests and full reporting of outcomes
☐	☒	Estimates of effect sizes (e.g. Cohen's *d*, Pearson's *r*), indicating how they were calculated

Our web collection on statistics for biologists contains articles on many of the points above.

Software and code

Policy information about availability of computer code

Data collection	Tucker–Davis system III hardware and software (TDT, Alachua, FL) for ABR testing; Rotarod apparatus (Ajanta Inc., India) for rotarod test; Zeiss LSM 510 confocal microscope for immunofluorescent figures, Zeiss Axioskop 2 Fs plus for resin section figures; Photoshop cc2018 for imaging.
Data analysis	GraphPad prism 5.0

For manuscripts utilizing custom algorithms or software that are central to the research but not yet described in published literature, software must be made available to editors/reviewers. We strongly encourage code deposition in a community repository (e.g. GitHub). See the Nature Research guidelines for submitting code & software for further information.

Data

Policy information about availability of data

All manuscripts must include a data availability statement. This statement should provide the following information, where applicable:

- Accession codes, unique identifiers, or web links for publicly available datasets
- A list of figures that have associated raw data
- A description of any restrictions on data availability

All relevant data in the manuscript and the supplementary materials are available. The source data underlying Figs. 3d, 5c, 6b–d, 7a–d, and Supplementary Figs.2b–h, 4d, 5a–d, 7d, and 9a–c are provided as a Source Data file (https://doi.org/10.6084/m9.figshare.12287774).Requests for materials should be addressed to Xi Lin, PhD (xlin2@emory.edu).

Field-specific reporting

Please select the one below that is the best fit for your research. If you are not sure, read the appropriate sections before making your selection.

- [x] Life sciences
- [ ] Behavioural & social sciences
- [ ] Ecological, evolutionary & environmental sciences

For a reference copy of the document with all sections, see nature.com/documents/nr-reporting-summary-flat.pdf

Life sciences study design

All studies must disclose on these points even when the disclosure is negative.

Sample size	No effect size was predetermined. All the experiments were performed at least three times. For statistical comparison, three replicated or more biological experiments were performed. The two-sided Student's t test was used to analyze ABR thresholds, amplitude of peak I wave, thickness of stria vascularis, cross-sections of the semicircular canals, outer diameters of bony SSCC, circling behavior, rotarod and swim test results, body weight growth, offspring birth and survival rates.
Data exclusions	Any damaged cochlea during the surgery or baby death after surgery was excluded from subsequent studies.
Replication	All experiments were repeated at least three times and we found similar results with no significant difference in terms of numbers or values.
Randomization	The alloccation was random.
Blinding	The vestibular function test videos were reviewed and scored by an observer who was blinded to the genotype and treatment and who was also not involved with the initial video recording.

Reporting for specific materials, systems and methods

We require information from authors about some types of materials, experimental systems and methods used in many studies. Here, indicate whether each material, system or method listed is relevant to your study. If you are not sure if a list item applies to your research, read the appropriate section before selecting a response.

Materials & experimental systems

n/a	Involved in the study	
[ ]	[x]	Antibodies
[ ]	[x]	Eukaryotic cell lines
[x]	[ ]	Palaeontology
[ ]	[x]	Animals and other organisms
[x]	[ ]	Human research participants
[x]	[ ]	Clinical data

Methods

n/a	Involved in the study	
[x]	[ ]	ChIP-seq
[x]	[ ]	Flow cytometry
[x]	[ ]	MRI-based neuroimaging

Antibodies

Antibodies used	The following antibodies were used: anti-GFP (Thermo Fisher, MA5-15256) , anti-Kcne1 (Mybiosource, MBS8503082) , anti-Myo7A (Proteus bioscience, cat#26-6790) , anti-NF200 (Invitrogen, cat#13-1300); isothiocyanate-conjugated phalloidin (Sigma-Aldrich, cat#P1951); Goat anti-Rabbit IgG (H+L) Highly Cross-Adsorbed Secondary Antibody, Alexa Fluor 488 (Invitrogen, cat# A11034), Goat anti-Rabbit IgG (H+L) Highly Cross-Adsorbed Secondary Antibody, Alexa Fluor Plus 555(Invitrogen, cat#A32727) .
Validation	All the antibodies are validated by the manufacturers.

Eukaryotic cell lines

Policy information about cell lines

Cell line source(s)	The HEK293T were purchased from the American Type Culture Collection (Manassas, VA, USA).
Authentication	The HEK293T cell line was not authenticated.
Mycoplasma contamination	The HEK293T cell line has been tested for mycoplasma contamination routinely.
Commonly misidentified lines (See ICLAC register)	*Name any commonly misidentified cell lines used in the study and provide a rationale for their use.*

Animals and other organisms

Policy information about studies involving animals; ARRIVE guidelines recommended for reporting animal research

Laboratory animals	Kcne1 KO mice (129-Kcne1tm1Sfh /J) and WT mice (129S1/SvlmJ) of both sexes in an estimated 50:50 ratio were used in this study. P0-3, P30, P60, P90, P120, P150 and P180 mice were used.
Wild animals	This study did not invovle wild animals.
Field-collected samples	The study did not involve samples collected from the field.
Ethics oversight	The Animal use protocols were approved by the Emory IACUC.

Note that full information on the approval of the study protocol must also be provided in the manuscript.
