# Supplementary material for: Gene therapy via canalostomy approach preserves auditory and vestibular functions in a mouse model of Jervell and Lange-Nielsen syndrome type 2
Source: Nat Commun. 2021 Jan 29;12:697. doi: 10.1038/s41467-020-20808-7 (PMC7846845; doi:10.1038/s41467-020-20808-7)
Supplement: Supplementary file 10 — Description of Additional Supplementary Files [file 41467_2020_20808_MOESM10_ESM.pdf]

Title: Supplementary Video 1:
Description: A demonstration showing surgical procedure for inner ear gene therapy via canalostomy approach in a neonatal Kcne1-/- mouse.

Title: Supplementary Video 2:
Description: Circling behavior and demonstration of test for an untreated Kcne1-/- mouse at P30.

Title: Supplementary Video 3:
Description: Circling test in a Kcne1-/- mouse from the high-dose-treated group tested at P30.

Title: Supplementary Video 4:
Description: Circling test showing a Kcne1 WT mouse examined at P30.

Title: Supplementary Video 5:
Description: Swimming test in a Kcne1 WT mouse examined at P30.

Title: Supplementary Video 6:
Description: Swimming test in an untreated Kcne1-/- mouse examined at P30.

Title: Supplementary Video 7:
Description: Swimming test in a high-dose-treated Kcne1-/- mouse examined at P30.
